# Cardiac protection by pirfenidone after myocardial infarction: a bioinformatic analysis

**DOI:** 10.1038/s41598-022-08523-3

**Published:** 2022-03-18

**Authors:** Alberto Aimo, Oriol Iborra-Egea, Nicola Martini, Carolina Galvez-Monton, Silvia Burchielli, Giorgia Panichella, Claudio Passino, Michele Emdin, Antoni Bayes-Genis

**Affiliations:** 1 Institute of Life Sciences, Scuola Superiore Sant’Anna, Piazza Martiri della Libertà 33, 56124 Pisa, Italy; 2 Cardiology Division, Fondazione Toscana Gabriele Monasterio, Pisa, Italy; 3 ICREC (Heart Failure and Cardiac Regeneration) Research Programme, Health Sciences Research Institute Germans Trias I Pujol (IGTP), Barcelona, Spain; 4 CIBER Cardiovascular, Instituto de Salud Carlos III, Madrid, Spain

**Keywords:** Computational biology and bioinformatics, Cardiology

## Abstract

Left ventricular (LV) remodeling after myocardial infarction (MI) is promoted by an intense fibrotic response, which could be targeted by the anti-fibrotic drug pirfenidone. We explored the relationship between protein modulation by pirfenidone and post-MI remodeling, based on molecular information and transcriptomic data from a swine model of MI. We identified 6 causative motives of post-MI remodeling (cardiomyocyte cell death, impaired myocyte contractility, extracellular matrix remodeling and fibrosis, hypertrophy, renin–angiotensin–aldosterone system activation, and inflammation), 4 pirfenidone targets and 21 bioflags (indirect effectors). Pirfenidone had a more widespread action than gold-standard drugs, encompassing all 6 motives, with prominent effects on p38γ-MAPK12, the TGFβ1-SMAD2/3 pathway and other effector proteins such as matrix metalloproteases 2 and 14, PDGFA/B, and IGF1. A bioinformatic approach allowed to identify several possible mechanisms of action of pirfenidone with beneficial effects in the post-MI LV remodeling, and suggests additional effects over guideline-recommended therapies.

## Introduction

In-hospital and 30-day mortality associated with ST-segment elevation myocardial infarction (STEMI) has steadily declined over the last decades given the implementation of percutaneous coronary intervention and medical therapy for neurohormonal antagonism^1^. This has been paralleled by a striking raise of patients who survived the acute event, but have an increased risk of heart failure (HF) and sudden chronic death^1^. The rate of HF hospitalization rate is 4.4% in the first year after STEMI, and around 1.0% per year thereafter^2^. In turn, patients who are hospitalized for HF during the first year have a risk of dying or being re-hospitalized for HF increased by 2- and 6-folds, respectively^2^.

Left ventricular (LV) remodeling consists in maladaptive changes in cardiac geometry and function developing as a result to the loss of viable myocardium and an increased wall stress. This process involves multiple mechanisms such as tissue resorption, extracellular matrix (ECM) degradation, deposition of granulation tissue and vessel formation, followed by scar maturation, fibrosis and hypertrophy in the remote myocardium. LV remodeling eventually leads to impaired ventricular function and HF^3^. Interventions able to prevent LV remodeling after STEMI are expected to improve the outcome of this condition.

Pirfenidone is a small synthetic molecule approved for idiopathic pulmonary fibrosis, a severe form of idiopathic interstitial pneumonias. It has antifibrotic as well as anti-oxidant and anti-inflammatory effects, which have been attributed to the inhibition of several growth factors, matrix metalloproteinases (MMP), and pro-inflammatory mediators, and possibly also an improvement of mitochondrial function and modulation of lymphocyte activation, although the exact mechanisms of action remain quite elusive^4,5^. Given that fibrosis plays a crucial role in several cardiac disorders, pirfenidone has been evaluated with interest as a possible cardiac protective medication, showing beneficial effects in animal models of different cardiac disorders, including LV remodeling after MI^6,7^. In the perspective of translating these findings into clinical trials, animal studies could be usefully implemented by a bioinformatic analysis evaluating the degree of overlap between the molecular pathways modulated by pirfenidone and those involved in post-MI remodeling in humans. In other words, if pirfenidone acts on several pathways leading to LV remodeling after MI, we may expect that pirfenidone therapy might prevent or relieve LV remodeling.

The first aim is to assess the functional relationship between the targets of pirfenidone and post-MI remodeling. The second aim is to identify the potential mechanisms of action of pirfenidone in the setting of post-MI remodeling. The third aim is to compare the molecular effects of pirfenidone and neurohormonal antagonists on post-MI remodeling.

## Methods

### Proteins involved in post-MI remodeling or modulated by pirfenidone

Articles published over the last 10 years on the molecular pathophysiology of post-MI remodeling were searched in PubMed on October 7, 2020 with the following keywords: post[title] and (infarct*[title] or stroke[title]) and (myocardial[title] or cardiovascular[title] or cardiac[title]) AND (pathophysiology[Title/Abstract] OR pathogenesis[Title/Abstract] OR molecular[Title/Abstract])). If the involvement of a protein candidate in post-MI remodeling was not well established, an additional PubMed search was performed, including all protein names according to UniProtKB.

To characterize the molecular effects of pirfenidone, we searched the European Medicines Agency: European Public Assessment Report, the Food and Drug Administration: Multidisciplinary review and Chemistry review, product monograph, and databases such as Drugbank, Reactome, MINT and BioGrid, as well as Anaxomics’ internal database^8^. We identified pirfenidone targets (i.e., proteins with a reported physical interaction with pirfenidone) and bioflags (proteins modulated by pirfenidone without a direct interaction). All this information was compiled into a framework database to feed the mathematical models.

### Post-MI gene expression data in swine

The Gene Expression Omnibus^9^ and Array Express^10^ public repositories were searched for gene expression data on post-MI remodeling with the following query (on December 9, 2020): post[ALL FIELDS] and (infarct*[ALL FIELDS] or stroke[ALL FIELDS]) and (myocardial[ALL FIELDS] or cardiovascular[ALL FIELDS] or cardiac[ALL FIELDS]). Studies in humans (*Organism: Homo sapiens*) reporting expression or protein arrays (*Series type: Expression profiling by array* and *protein profiling by protein array*) were searched. No gene expression datasets from myocardial tissue biopsies could be found. Our group previously employed microarray gene expression profiling of porcine cDNA to compare myocardial gene expression 1, 4, and 6 weeks after surgically induced MI and in sham-operated controls^11^. The swine transcriptomics were translated to their human equivalents via Reciprocal Best Hits (RBH) with BLAST and Gene Name Correspondence and the InParanoid database^12^. When RBH were not found, we used the UniProt entry for the human protein with a matching gene name as a correspondence.

Microarray data was processed using the GEO2R tool^9^, and processed using the neqc method^13^ and Linear Models for Microarray Analysis^14^. We only considered genes with an adjusted p value of < 0.01 (Benjamini–Hochberg false discovery rate), and log(fold-change) > 0.25. For introduction into the protein network, gene information was mapped one-to-one with proteins. In the end, 4,737 proteins were included.

This information was used as experimental reference to model the algorithms and contrast the in silico findings to ensure their adhere to experimental evidence.

### Generation of mathematical models

Molecular descriptions of input‐output signals were obtained through manual literature mining and the KEGG, MINT, REACTOME, BIOGRID and DrugBank databases. The inputs included information about drugs, pathologies, and protein/gene relationships that could inhibit or activate at least one node of the network. The outputs were microarray data regarding up‐ or down‐regulated genes/proteins, and clinical information. Mathematical algorithms were generated to trace connections and elucidate their mechanisms. The models were then used to determine the weight of each relationship. To optimize the system, we used a Montecarlo-based approach^15^, and another based on information from network topology^16^.

### Biological map linking disease effectors and pirfenidone targets

The therapeutic performance mapping system technology employs 2 different and complementary strategies. The artificial neural network (ANN) strategy can identify relationships among network regions (generalization)^17^, allowing to infer the likelihood of a relationship between ≥ 2 sets of proteins. In this case, we tested each protein against the post-MI remodeling signature. Next, the model is validated using different data sets from the literature and databases. This system attempts to find the shortest distance between the 2 protein sets, generating a list of proteins ranked by their association with disease pathophysiology. A model-based mechanism of action (MoA) of pirfenidone was then created to further analyze drug efficacy. The second strategy is the sampling method, which is applied once a key region of the protein map has been identified using ANNs or is suggested by experimental work. Once a response has been identified and linked to a specific stimulus through ANNs, sampling methods enable analysis of the MoA, and elucidation of the underlying relationship.

### Unsupervised comparison between pirfenidone and other drugs

ANN scores were calculated for angiotensin converting enzyme inhibitors (ACEi; target gene *ACE*, Uniprot P12821), angiotensin receptor blockers (ARB; target gene *AGTR1*, Uniprot P30556), beta-blockers (target gene *ADRB1*, Uniprot P08588), and mineralocorticoid receptor antagonists (MRA; target gene *NR3C2*, Uniprot P08235). We constructed a protein–protein interaction (PPI) network that contains all post-MI effectors to investigate the biological interactions of ACEi, ARB, beta-blockers, and MRA targets. We used the String platform, where each PPI is annotated with one or more “scores”^18^. All scores rank from 0 to 1, with 1 being the highest possible confidence in judging the interaction as true. Here we used a minimum score of 0.9 to investigate how ACEi/ARB, beta-blockers, and MRA targets interact with our molecular characterization of post-MI remodeling, which includes 222 proteins (Supplemental Table 1). Unsupervised clustering was performed using a K-Means approach, with the number of clusters (K = 10) determined through the elbow method.

### Binding affinity simulation

To model the binding affinity of Pirfenidone towards both predicted direct targets (Furin and MAPK12), we used the kdeep platform^19^. Pirfenidone sdf file was obtained from (http://realtime.molinstincts.com/). MAPK12 crystal structure (PDB file) was extracted from the protein databank (reference code 6UNA)^20^. Furin crystal structure was only available for mus musculus^21^. The Phyre2 web portal was used to create a 3D render of the human Furin protein^22^. Four templates were selected to model the protein based on heuristics to maximize confidence, percentage identity and alignment coverage. Here, 93% of residues were modelled at > 90% confidence. To compare the binding affinities of our docking simulation with current known ligands, we performed a screen of 72 inhibitors against 456 human kinases has been performed using quantitative data derived using DiscoveRx KINOMEscan platform^23,24^.

## Results

### Characterization of post-MI remodeling, pirfenidone targets and bioflags

The causative motives (i.e., pathophysiological mechanisms causally linked with the development of post-MI remodeling) were: cardiomyocyte cell death, impaired myocyte contractility, ECM remodeling and fibrosis, hypertrophy, renin–angiotensin–aldosterone system (RAAS) activation, and inflammation (Table 1). A total of 217 non-duplicated effectors (i.e., proteins functionally associated with each motive) were identified (Supplemental Table 1), as well as 4 targets and 21 bioflags (indirect effectors) of pirfenidone (Supplemental Table 2).

**Table 1:** Causative motives identified as involved in post-myocardial infarction remodeling.

Motive name	Number of proteins
ECM remodelling and fibrosis	53
Hypertrophy	51
Cardiomyocyte cell death	49
Impaired cardiomyocyte contractility	41
Inflammation	30
RAAS activation	23

*ECM* extracellular matrix, *RAAS* renin–angiotensin–aldosterone system. The complete list of proteins is reported in Supplemental Table 1.

### Mechanistic relationship between pirfenidone and post-MI remodeling

When evaluating all the motives simultaneously, and considering just pirfenidone targets, the level of mechanistic relationship of pirfenidone with post-MI remodeling was low, and cardiomyocyte cell death seemed to be the most affected motive. An intermediate degree of relationship between each pirfenidone target and post-MI remodeling was found. When considering the whole bioflag profile, pirfenidone showed a medium-to-high degree of relationship with all the pathophysiological motives involved in post-MI remodeling (Table 2).

**Table 2:** Prediction of functional relationship between pirfenidone targets and bioflags and post-myocardial infarction (MI) remodelling.

Treatment, targets/bioflags	Likelihood of association with post-MI remodeling	Most affected motive	Likelihood of association with motive
Pirfenidone, full target profile	+	Cardiomyocyte cell death	+++
Pirfenidone, furin	++	RAAS activation	+++
Pirfenidone, PAI-1	++	ECM remodeling and fibrosis	+
Pirfenidone, MAPK12	++	Cardiomyocyte cell death	+++
Pirfenidone, GLI2	++	Cardiomyocyte cell death	+++
Pirfenidone, full target and bioflag profile	++	All motives	++
**Pirfenidone bioflags with maximum value for each motive**			
GSK3B	+++	Cardiomyocyte cell death	+++
TGFB1	+++	ECM remodeling and fibrosis	+++
FN1	++	ECM remodeling and fibrosis	+++
JUN	++	Hypertrophy	+++
ACE	++++	RAAS activation	+++
IFNG	+++	Inflammation	+++

*ACE* angiotensin-converting enzyme, *FN1* fibronectin, *GLI2* GLI family zinc finger 2, *GSK3B* glycogen synthase kinase-3 beta, *IFNG* interferon gamma, *MAPK12* mitogen activated protein kinase-12, *PAI-1* plasminogen activator inhibitor 1, *TGFB1* transforming growth factor beta 1.

### Mechanisms of action of pirfenidone in post-MI remodeling

**Figure 1:**
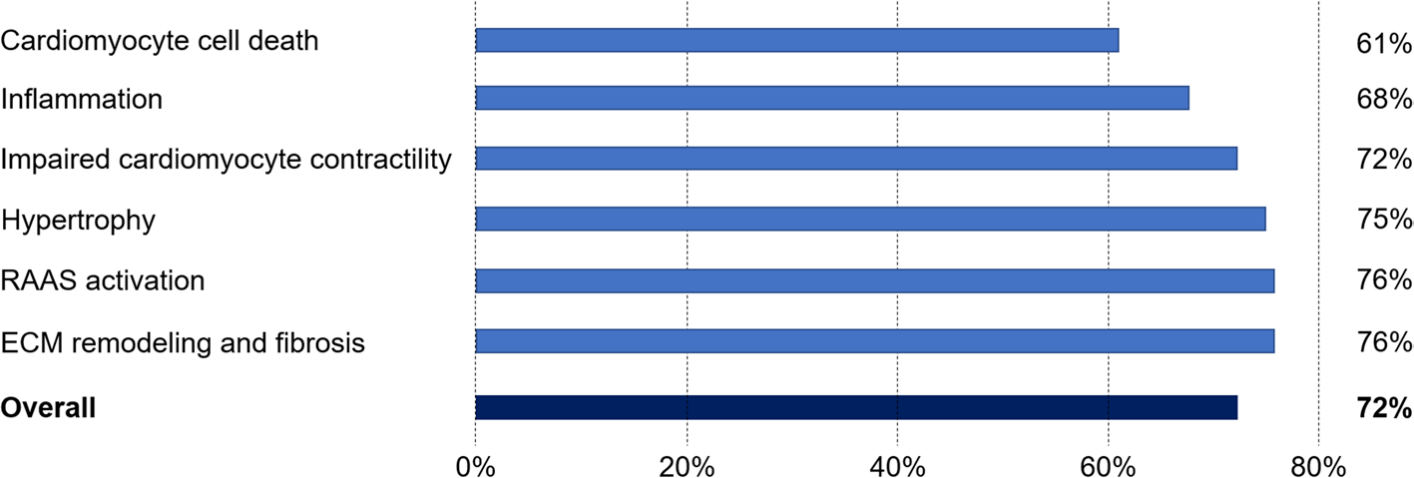
Likelihood of the effect of pirfenidone on post-MI remodeling and on single motives. *ECM* extracellular matrix, *RAAS* renin–angiotensin–aldosterone system.

**Figure 2:**
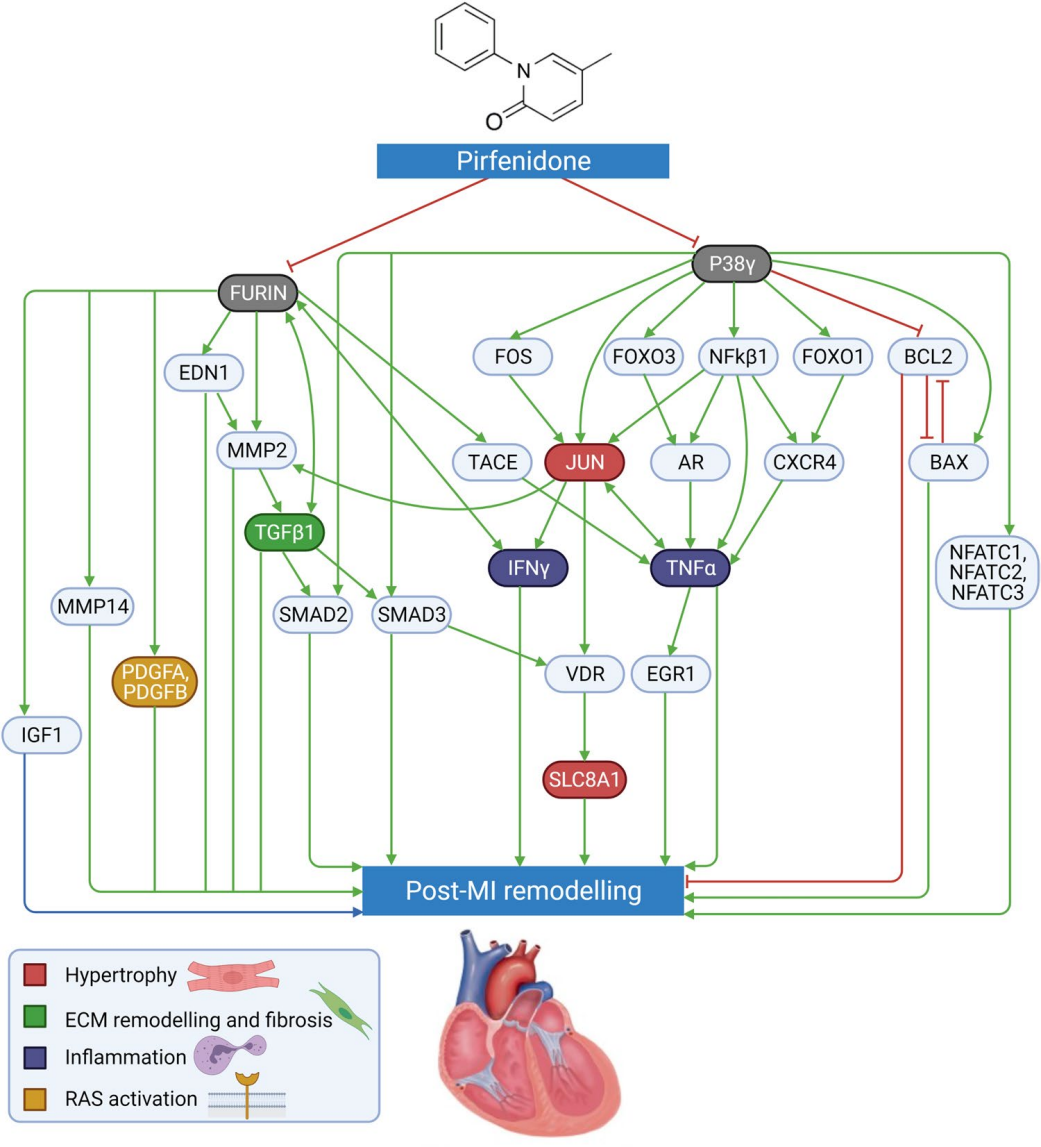
Proposed protein network mediating the effects of pirfenidone on post-myocardial infarction (MI) left ventricular remodeling. See text for details and acronyms.

As displayed in Fig. 1, pirfenidone had a strong impact on all 6 motives involved in post-MI remodeling. In detail, 72% of the protein alterations found in post-MI remodeling were predicted to be reversed by pirfenidone treatment. Unlike in the ANN-based analyses (which focus on direct mechanistic interactions), sampling-based models predicted a lower modulation of the cardiomyocyte cell death motive (61%) compared to the other motives. The most likely mechanisms of action of pirfenidone in the post-MI setting were identified, and visually represented in Fig. 2; the proteins reported in the nodes of the graph are listed in Supplemental Table 3.

**Figure 3:**
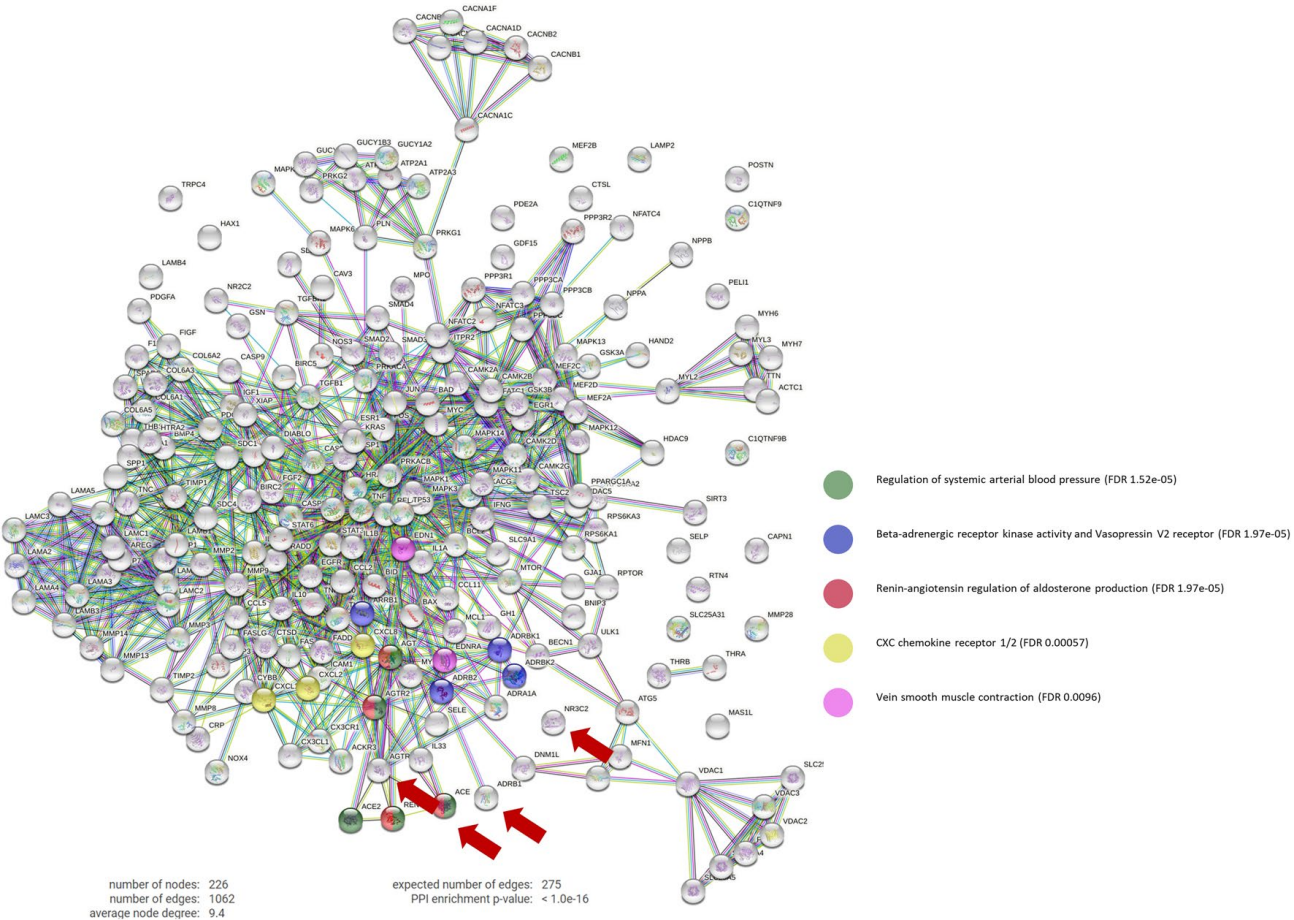
Targets of pirfenidone (black arrows) vs. targets of guideline-recommended therapies (red arrows).

Mitogen activated protein kinase-12 (MAPK12) and furin seemed to be the main mediators of pirfenidone effects, with a less prominent role for the remaining targets (GLI family zinc finger 2 [GLI2] and plasminogen activator inhibitor 1 [PAI-1]). Several pirfenidone bioflags were modulated in the mechanistic representation: JUN, tumor necrosis factor-α (TNFα), transforming growth factor-β1 (TGF-β1), platelet-derived growth factors A and B (PDGFA/PDGFB), interferon-γ (IFN-γ) (Fig. 3).

### Pirfenidone has a high binding affinity for MAPK12 and furin

**Figure 4:**
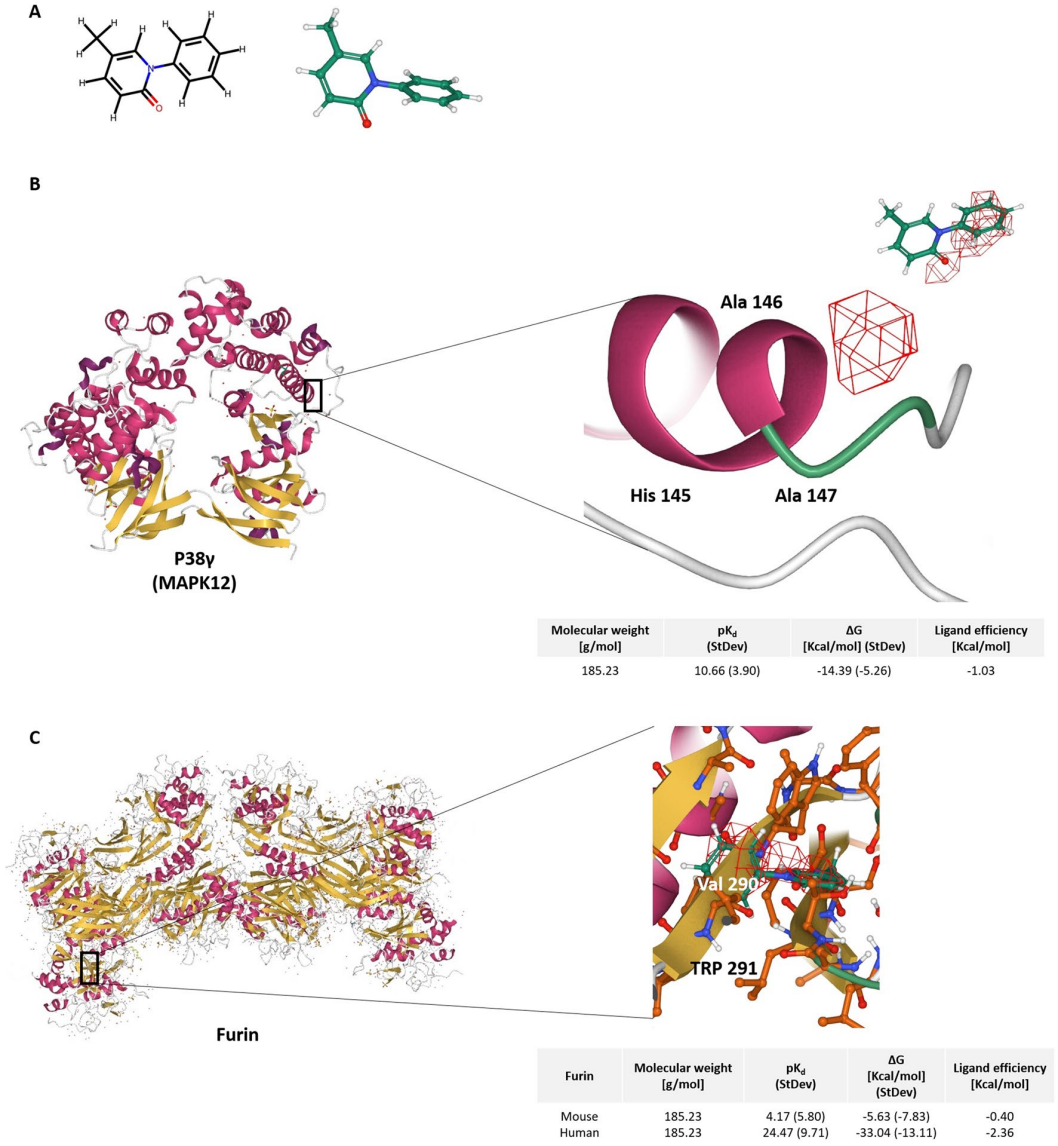
3D Models of pirfenidone binding to MAPK12 and furin. (**A**) 2D and 3D structure of pirdenidone; (**B**) Pirfenidone docking site with MAPK12. Pirfenidone seems to attach more strongly at the Ala146 residue in the conserved region and near active binding sites; (**C**) Pirfenidone docking site with furin. Pirfenidone has a much higher binding affinity for the human model of furin than the mouse model. Pirfenidone appears to bind at the catalytic domain at the Val 290 residue.

3D models of pirfenidone binding to MAPK12 and furin have been constructed to explore binding affinity properties (Fig. 4). Pirfenidone appears to bind more strongly at the Ala146 residue in the conserved region of MAPK12, near other active binding sites, while it seems to have a much higher binding affinity for the human model of furin than the mouse model, bind at the catalytic domain at the Val 290 residue.

The 10 most potent ligands for MAPK12 and all 4 known inhibitors of furin are shown in Table 3, compared with Pirfenidone. Doramapimod (as research code BIRB 796) is the best-known MAPK12 inhibitor, although it is not specific to MAPK12. Doramapimod has a pKd value of 8.5 and has been assessed in Phase II clinical trials for plaque-type psoriasis and rheumatoid arthritis but has not progressed beyond Phase II. MI-1148 is a synthetic organic compound specifically design to inhibit furin, with a pKd value of 11.3 and has been tested as an anti-infective agent.

**Table 3:** List of the 10 most potent ligands for MAPK12 and all known inhibitors of Furin binding affinities compared with pirfenidone.

	Ligand	Type	pKd value
MAPK12	Pirfenidone*	Inhibitor	10.66
	Doramapimod	Inhibitor	8.5
	AST-487	Inhibitor	7.5
	Staurosporine	Inhibitor	7.4
	Lestaurtinib	Inhibitor	6.4
	Tamatinib	Inhibitor	6.4
	Foretinib	Inhibitor	6.4
	SB203580	Inhibitor	5.8
	Linifanib	Inhibitor	5.7
	Ruboxistaurin	Inhibitor	< 5.5
	Erlotinib	Inhibitor	< 5.5
Furin	Pirfenidone*	Inhibitor	24.47
	MI-1148	Inhibitor	11.3
	Phenylacetyl-Arg-Val-Arg-4-Amidinobenzylamide	Inhibitor	9.1
	Peptide 18	Inhibitor	8.4
	Furin inhibitor peptide	Inhibitor	7.6

In our analysis, pirfenidone has a predicted better binding affinity to MAPK12 (10.66 vs 8.5 doramapimod) and furin (24.47 vs 11.3 MI-1148) than any reported experimental inhibitor.

### Pirfenidone vs. other drugs for MI

According to the obtained ANNs scores, the other compared drugs may modulate different pathophysiological motives more specifically, while pirfenidone (when considering the totality of biotargets and bioflags) seemed to impact on all motives, and then to have potentially a broader effect on post-MI remodeling (Table 4).

**Table 4:** Effects of pirfenidone and guideline-recommended drugs on post-myocardial infarction (MI) remodeling and its single motives.

Treatment	Post-MI remodelling	Cardiomyocyte cell death	Impaired cardiomyocyte contractility	ECM remodeling and fibrosis	Hypertrophy	Inflammation	RAAS activation
Pirfenidone (only targets)	34%	70%	24%	14%	19%	25%	16%
Pirfenidone (targets + bioflags)	49%	71%	73%	71%	77%	70%	71%
ACEi	81%	5%	3%	72%	77%	3%	77%
ARB	78%	57%	31%	33%	86%	36%	85%
Beta-blockers	68%	24%	74%	7%	73%	3%	81%
MRA	74%	72%	12%	12%	82%	5%	3%

*ACEi* angiotensin-converting enzyme inhibitors, *ARB* angiotensin receptor blockers, *ECM* extracellular matrix, *MRA* mineralocorticoid receptor antagonists, *RAAS* renin–angiotensin–aldosterone system.

Unsupervised PPI analyses showed ACEis and ARBs exerted their function upon the RAS system as well as modulate modestly pro-inflammatory pathways through CXC receptors in neutrophiles (which bind to interleukin [IL]-8 and IL-6). Beta-blockers modulate the beta-adrenergic receptor kinase activity, but also venous smooth muscle contraction, which in turn is also related to the RAS pathway. Finally, MRAs (and specifically NR3C2) did not interact with any post-MI effector in our network. Accordingly, ACE1 (ACEi), AGTR1 (ARB) and ADRB1 (beta-blockers) cluster together, including the proteins comprising their downstream effects, but NR3C2 (MRA) gets clustered separately.

Compared to the targets of ACEi/ARB, beta-blockers and MRA, pirfenidone targets (furin, MAPK12, PAI-I and GLI-2) are much more interconnected with the post-MI effector network. GLI2 and MAPK12 modulation effects a downstream signaling cascade involving glycogen synthase kinase 3 beta (GSK3B) and cyclic adenosine monophosphate (cAMP)-dependent protein kinase catalytic subunits. Individually, furin directly modulates ECM remodeling through matrix metalloproteinase-14 (MMP14) and other downstream MMPs and several laminin subunits. SerpinE1, is also involved in MMPs modulation through tissue inhibitor of metalloproteinase-1 (TIMP1), but also regulates cell differentiation, migration and cell death pathways. Furin and SerpinE1 combined, synergistically modulate the advanced glycation end products (AGE)/ receptor for AGEs (RAGE) signaling pathway.

## Discussion

A comprehensive literature search allowed to identify 6 causative motives of post-MI remodeling (cardiomyocyte cell death, impaired myocyte contractility, ECM remodeling and fibrosis, hypertrophy, RAAS activation, and inflammation), 4 pirfenidone targets and 21 bioflags. When considering both targets and bioflags, pirfenidone showed a broad relationship encompassing all 6 motives. Finally, a limited degree of overlap was found between pirfenidone and guideline-recommended drugs (ACEi/ARB, beta-blockers, and MRA), supporting an additive role of pirfenidone in this setting.

Pirfenidone is an established drug for idiopathic pulmonary fibrosis, and was recently demonstrated to reduce LV fibrosis (assessed by cardiac magnetic resonance as extracellular volume) in patients with heart failure with preserved ejection fraction^25^. In this study we investigated the conceptual framework for pirfenidone as an effective drug after MI, thanks, but not limiting to, its antifibrotic activity.

MAPK12 and furin are the most prominent mediators of the effects of pirfenidone on post-MI remodeling. MAPK12, also known as p38γ, is a serine/threonine kinase that phosphorylates a wide range of proteins^26^. For example, it inactivates BCL2, thus inducing cell apoptosis^27^. MAPK12 is also reported to enhance BAX, which has pro-apoptotic effects^28^. Nuclear factors of activated T-cells (NFAT) signaling is involved in cardiac hypertrophy. Inhibited MAPK12 is not able to stimulate the activation of NFATC signaling, therefore its inhibition may relieve hypertrophy^29^. Forkhead box O protein 1 (FOXO1) is activated by MAPK12^30^, and regulates C-X-C Motif Chemokine Receptor 4 (CXCR4), which may stimulate fibroblast proliferation or inflammation^31^. Downstream of CXCR4, TNFα production is stimulated^32^. TNFα expression is increased after MI, promoting the progress of cardiac remodeling by inducing the secretion of apoptosis-related proteins and inflammatory factors^33^. Early growth response protein 1 (EGR1) can be upregulated by TNFα^34^, contributing to the maladaptive hypertrophy, HF, and arrhythmias^35^. By blocking this pathway, pirfenidone may modulate cardiomyocyte hypertrophy, interstitial fibrosis and inflammation.

Pirfenidone might also inhibit upstream mediators to this pathway. Activator protein 1 (AP-1) is a transcription factor that can be activated by p38 MAPK and regulate cardiomyocyte hypertrophy. TNFα has also been reported to upregulate c-JUN, which upregulates IFN-γ^36^. IFN-γ is a cytokine with multiple immunologic effects, including macrophage activation, regulation of the Th1/Th2 balance, cellular proliferation and apoptosis^37^. IFN-γ blockade by pirfenidone may reduce inflammation and cardiac fibrosis. IFN-γ has also been reported to increase transcript levels of furin^38^. Therefore, pirfenidone seems to modulate the effect of furin both directly and indirectly through MAPK12 signaling. Furin is a ubiquitous endoprotease that cleaves and activates a wide range of proproteins including TGF-β1^39^, insulin-like growth factor 1 (IGF-1)^40^, disintegrin and metalloproteinase domain-containing protein 17 (TACE)^41^, endothelin 1 (EDN1)^42^, MMP2^43^, MMP14^44^, and PDGF^45^. TGF-β1 is produced within injured tissues, inhibits immune cell proliferation and stimulates fibroblasts to synthesize ECM proteins^46^. Besides its role in inflammation and fibrosis, TGF-β1 blunts cardiomyocyte contractility^47^ and hypertrophic response^48^. The effects of pirfenidone on multiple processes might be partially attributed to these multiple effects of TGF-β1. PDGFs are involved in organ fibrosis by stimulating the proliferation, migration and survival of myofibroblasts, responsible for the deposition of ECM proteins^49^, and blockade of PDGFs by pirfenidone may then exert beneficial effects. Proteolytic enzymes such as MMP2 released by cells within injured tissues can increase collagen deposition, thus contributing to cardiac fibrotic remodeling^50^. Moreover, proteins degraded by MMP2 contribute to the depressed contractility after MI^51^. The ECM acts as a store of inactive TGF-β1, and MMP2 can promote TGF-β1 activation by proteolytically releasing them from the inactive ECM-bound complex^52^. Overall, pirfenidone´s inhibition of furin might affect these downstream effectors, thus ameliorating post-MI remodeling. By reducing the activity of furin, active EDN1 levels are also reduced. EDN1 is found to be correlated with cardiac remodeling and hypertrophy, and can cause coronary vasoconstriction, thereby reducing myocardial perfusion and increasing infarct size^53^. TACE is a metallopeptidase cleaved and activated by furin, which functions as a TNFα converting enzyme^54^. Therefore, pirfenidone might downregulate TACE activation, modulating the inflammation, tissue remodeling and dysfunction mediated by TNFα. Pirfenidone may also decrease the levels of IGF1 through its inhibitory effect on furin and MAPK12, and this could modulate myocyte cellular hypertrophy^55^. In summary, pirfenidone seems to modulate all the pathophysiologic processes related to post-MI remodeling by blocking the activity of MAPK12 and furin.

We also investigated the binding affinity of pirfenidone to both targets (MAPK12 and furin) and compared it to those of other known, tested, ligands. The Gibbs free energy (G) model, as well as the pKd values were used as indicators of good binding affinities (Fig. 4). In the first case, if ΔG is negative, the reaction does not require external energy to occur, and the larger the negative number, the easier is to pair. The latter value is the negative logarithm of K*d* (the equilibrium dissociation constant between the ligand and the protein), and the larger the positive pK*d* values, the higher is the affinity between the pair. With this experiment, it seems apparent that pirfenidone has high binding affinities for both proteins, with predicted binding values much larger than with other reported compounds, which indicates a clear biological interaction and reinforces the results of the machine learning approach.

Pirfenidone also stimulates voltage-gated calcium channels (via cAMP-dependent protein kinase)^56^, excitation–contraction coupling, and contractility. Moreover, it also exerts antioxidant effects and inhibits the Na/Ca exchanger^57^. The latter, combined with the current view that the sodium/calcium exchanger 1 (SLC8A1) is expected to mediate effects of pirfenidone on post-myocardial infarction remodeling (Supplemental Table 3), could help link calcium homeostasis effects with the benefits in post-MI LV. Another potential contributor is the alfa 1C calcium channel (CACNA1C), as shown in Fig. 3.

Interestingly, pirfenidone targets were much more entrenched in the protein network than targets of all guideline-recommended drugs. Through GLI2 and MAPK12 modulation, a downstream signaling cascade involving GSK3B and cAMP-dependent protein kinase catalytic subunits could improve myocyte contractility and electric depolarization by intracellular calcium handling. Individually, furin directly modulates MMP14 (and other MMPs), and proteolytic processing of signaling molecules by MMPs, particularly transmembrane MMPs, may facilitate ECM accumulation^58^. It is also highly involved in ECM remodeling through the regulation of several laminin subunits. SerpinE1, is also involved in MMP modulation, but also regulates cell differentiation, migration and cell death. More importantly, both furin and serpinE1 are key modulators of the AGE/RAGE signaling pathway. High levels of glucose nonenzymatically react with long-lived proteins, such as collagen, to form AGEs. AGE-modified collagen increases matrix stiffness making it resistant to hydrolytic turnover, resulting in an accumulation of ECM proteins. AGEs account for many of the diabetic cardiovascular complications through their engagement of RAGE. AGE/RAGE activation stimulates the secretion of numerous profibrotic growth factors, promotes increased collagen deposition leading to tissue fibrosis, as well as increased RAGE expression^59^.

It is important to note that, although Didiasova et al.^60^ demonstrated pirfenidone’s downregulation effect upon GLI2, this is not definitive proof of a direct interaction between the drug and GLI2, and there could be several mediators in between the signaling cascade. Practically, this has no major impact on our analysis, as our models explored the effect of GLI2 downregulation, independently of the nature of the downregulation. Nonetheless, this is interesting because our models found a very limited role for GLI2 overall, and due to the relative novelty of pirfenidone post-MI, the limited information about GLI2 and PAI-1 targets could underestimate their role.

Interestingly, pirfenidone has been clearly demonstrated to have an important role in oxidative stress^61,62^. Indeed, one of the first effects described for pirfenidone was a reactive oxygen species scavenging activity^63^. However, our analysis has not found this effect as meaningful in this scenario. Our analysis was intended to analyze specifically the molecular mechanism of action of pirfenidone upon adverse cardiac remodeling, which is a discrete aspect of the spectrum of affected processes post-MI. Importantly, this is not to say that oxidative stress does not play a role in the mechanism of action of pirfenidone, which could certainly be important as demonstrated in several publications. However, our analysis indicate that oxidative stress may not be the main effect of pirfenidone to directly ameliorate adverse cardiac remodeling.

This could be due to several reasons. First, our models worked out the main mechanism of action in the heart, which was suggested to be mediated by Furin and P38γ, and only explored in depth this pathway. Here, oxidative stress could be mediated by a secondary mechanism independent of the heart or these key proteins, and thus our models could not fully incorporate it. Moreover, oxidative stress could be more important to a different outcome than the one established in our models. Our analysis found the most probable path from the input (pirfenidone) to the output (cardiac remodeling post-MI), restricted by the high throughput and clinical data. Thus, changing the output under consideration could have different implications on the results.

Several limitations must be acknowledged. The information considered here has already been described or uploaded in public repositories. The data reported here is valid for post-MI remodeling and cannot be extrapolated to other cardiovascular pathologies. Further experimental validation is required to better understand the MoA of pirfenidone and its role in post-MI. Moreover, strong associations in our models (for MAPK12 and Furin) could be masking deeper roles of other targets and bioflags that still have relatively few data to input in the model.

In conclusion, a bioinformatic approach allowed to identify several possible mechanisms of action of pirfenidone with beneficial effects in the post-MI LV remodeling, and suggests additional effects over standard guideline-recommended therapies. These findings support clinical studies evaluating the beneficial effects of pirfenidone in patients with MI.

## Supplementary Information

Supplementary Tables.
