# Supplementary material for: Cardiac protection by pirfenidone after myocardial infarction: a bioinformatic analysis
Source: Sci Rep. 2022 Mar 18;12:4691. doi: 10.1038/s41598-022-08523-3 (PMC8933518; doi:10.1038/s41598-022-08523-3)
Supplement: Supplementary file 1 — Supplementary Tables. [file 41598_2022_8523_MOESM1_ESM.docx]

**Supplemental material**

**Cardiac protection by pirfenidone after myocardial infarction: a bioinformatic analysis**

Alberto Aimo^1,2*^, Oriol Iborra-Egea^3,4*^, Nicola Martini^2^, Carolina Galvez-Monton^3^, Silvia Burchielli^2^, Giorgia Panichella^1^, Claudio Passino^1,2^, Michele Emdin^1,2^, Antoni Bayes-Genis^3,4^

1. Institute of Life Sciences, Scuola Superiore Sant’Anna, Pisa, Italy; 2. Fondazione Toscana Gabriele Monasterio, Pisa, Italy; 3. ICREC (Heart Failure and Cardiac Regeneration) Research Programme, Health Sciences Research Institute Germans Trias i Pujol (IGTP); 4. CIBER Cardiovascular, Instituto de Salud Carlos III, Madrid, Spain. *These Authors equally contributed.

**Supplemental Table 1. Motives involved in post-myocardial infarction remodeling and corresponding protein effectors.**

| **Motives** | **Effector Protein (Name)** | **Effector Protein**  **(Short Name)** | **Effector**  **Protein (Uniprot)** | **Causative Effect**  **(1, -1)** | **Relevance (100-75)** | **References** |
| --- | --- | --- | --- | --- | --- | --- |
| ECM remodelling and fibrosis | Laminin subunit beta-4 | LAMB4 | A4D0S4 | 1 | 80 | [1] PMID: 24530674; [2] PMID: 19593942 |
| ECM remodelling and fibrosis | Collagen alpha-5(VI) chain | COL6A5 | A8TX70 | 1 | 80 | [1] PMID: 24072174; [2] PMID: 19593942 |
| ECM remodelling and fibrosis | Laminin subunit alpha-5 | LAMA5 | O15230 | 1 | 80 | [1] PMID: 24530674; [2] PMID: 19593942 |
| ECM remodelling and fibrosis | Coagulation factor XIII A chain | F13A1 | P00488 | -1 | 75 | [1] PMID: 23316962; [2] PMID: 15364610; [3] PMID: 18276618 |
| ECM remodelling and fibrosis | Angiotensinogen | AGT | P01019 | 1 | 75 | [1] PMID: 25752645; [2] PMID: 26562414 |
| ECM remodelling and fibrosis | Metalloproteinase inhibitor 1 | TIMP1 | P01033 | -1 | 85 | [1] PMID: 23316962 |
| ECM remodelling and fibrosis | Transforming growth factor beta-1 proprotein | TGFB1 | P01137 | 1 | 80 | [1] PMID: 25583678 |
| ECM remodelling and fibrosis | Interleukin-1 alpha | IL1A | P01583 | 1 | 75 | [1] PMID: 23806284; [2] PMID: 18535174 |
| ECM remodelling and fibrosis | Fibronectin | FN1 | P02751 | 1 | 80 | [1] PMID: 23316962; [2] PMID: 26498937 |
| ECM remodelling and fibrosis | Estrogen receptor | ESR1 | P03372 | -1 | 75 | [1] PMID: 24977106 |
| ECM remodelling and fibrosis | Interstitial collagenase | MMP1 | P03956 | 1 | 80 | [1] PMID: 24519465 |
| ECM remodelling and fibrosis | Endothelin-1 | EDN1 | P05305 | 1 | 75 | [1] PMID: 25595790 |
| ECM remodelling and fibrosis | Procathepsin L | CTSL | P07711 | 1 | 80 | [1] PMID: 21147810 |
| ECM remodelling and fibrosis | Laminin subunit beta-1 | LAMB1 | P07942 | 1 | 80 | [1] PMID: 24530674; [2] PMID: 19593942 |
| ECM remodelling and fibrosis | Thrombospondin-1 | THBS1 | P07996 | -1 | 80 | [1] PMID: 26498937; [2] PMID: 23316962 |
| ECM remodelling and fibrosis | 72 kDa type IV collagenase | MMP2 | P08253 | 1 | 90 | [1] PMID: 21671800; [2] PMID: 18535174 |
| ECM remodelling and fibrosis | Stromelysin-1 | MMP3 | P08254 | 1 | 90 | [1] PMID: 23806284; [2] PMID: 18535174 |
| ECM remodelling and fibrosis | Matrilysin | MMP7 | P09237 | 1 | 85 | [1] PMID: 23316962 |
| ECM remodelling and fibrosis | SPARC | SPARC | P09486 | 1 | 75 | [1] PMID: 23316962; [2] PMID: 15364610; [3] PMID: 18276618 |
| ECM remodelling and fibrosis | Osteopontin | SPP1 | P10451 | 1 | 75 | [1] PMID: 15364610; [2] PMID: 24072174 |
| ECM remodelling and fibrosis | Laminin subunit gamma-1 | LAMC1 | P11047 | 1 | 80 | [1] PMID: 24530674; [2] PMID: 19593942 |
| ECM remodelling and fibrosis | Collagen alpha-1(VI) chain | COL6A1 | P12109 | 1 | 80 | [1] PMID: 24072174; [2] PMID: 19593942 |
| ECM remodelling and fibrosis | Collagen alpha-2(VI) chain | COL6A2 | P12110 | 1 | 80 | [1] PMID: 24072174; [2] PMID: 19593942 |
| ECM remodelling and fibrosis | Collagen alpha-3(VI) chain | COL6A3 | P12111 | 1 | 80 | [1] PMID: 24072174; [2] PMID: 19593942 |
| ECM remodelling and fibrosis | Angiotensin-converting enzyme | ACE | P12821 | 1 | 80 | [1] PMID: 23316962 |
| ECM remodelling and fibrosis | Matrix metalloproteinase-9 | MMP9 | P14780 | 1 | 90 | [1] PMID: 23806284 |
| ECM remodelling and fibrosis | Amphiregulin | AREG | P15514 | 1 | 75 | [1] PMID: 32188578 |
| ECM remodelling and fibrosis | Metalloproteinase inhibitor 2 | TIMP2 | P16035 | -1 | 90 | [1] PMID: 23806284; [2] PMID: 23316962 |
| ECM remodelling and fibrosis | Gap junction alpha-1 protein | GJA1 | P17302 | -1 | 80 | [1] PMID: 23316962 |
| ECM remodelling and fibrosis | Syndecan-1 | SDC1 | P18827 | -1 | 80 | [1] PMID: 24519465 |
| ECM remodelling and fibrosis | Interleukin-10 | IL10 | P22301 | -1 | 80 | [1] PMID: 25583678 |
| ECM remodelling and fibrosis | Neutrophil collagenase | MMP8 | P22894 | 1 | 80 | [1] PMID: 24519465 |
| ECM remodelling and fibrosis | Laminin subunit alpha-2 | LAMA2 | P24043 | 1 | 80 | [1] PMID: 24530674; [2] PMID: 19593942 |
| ECM remodelling and fibrosis | Tenascin | TNC | P24821 | 1 | 75 | [1] PMID: 15364610; [2] PMID: 24072174 |
| ECM remodelling and fibrosis | Laminin subunit alpha-1 | LAMA1 | P25391 | 1 | 80 | [1] PMID: 24530674; [2] PMID: 19593942 |
| ECM remodelling and fibrosis | Syndecan-4 | SDC4 | P31431 | -1 | 80 | [1] PMID: 24519465 |
| ECM remodelling and fibrosis | Alpha-1A adrenergic receptor | ADRA1A | P35348 | -1 | 75 | [1] PMID: 16859660 |
| ECM remodelling and fibrosis | Metalloproteinase inhibitor 3 | TIMP3 | P35625 | -1 | 85 | [1] PMID: 21243368 |
| ECM remodelling and fibrosis | TGF-beta receptor type-2 | TGFBR2 | P37173 | 1 | 80 | [1] PMID: 22882904 |
| ECM remodelling and fibrosis | Collagenase 3 | MMP13 | P45452 | 1 | 80 | [1] PMID: 24519465 |
| ECM remodelling and fibrosis | Matrix metalloproteinase-14 | MMP14 | P50281 | 1 | 80 | [1] PMID: 17928585 |
| ECM remodelling and fibrosis | Laminin subunit beta-2 | LAMB2 | P55268 | 1 | 80 | [1] PMID: 24530674; [2] PMID: 19593942 |
| ECM remodelling and fibrosis | Mothers against decapentaplegic homolog 3 | SMAD3 | P84022 | 1 | 80 | [1] PMID: 22882904 |
| ECM remodelling and fibrosis | Mothers against decapentaplegic homolog 4 | SMAD4 | Q13485 | 1 | 80 | [1] PMID: 22882904 |
| ECM remodelling and fibrosis | Laminin subunit beta-3 | LAMB3 | Q13751 | 1 | 80 | [1] PMID: 24530674; [2] PMID: 19593942 |
| ECM remodelling and fibrosis | Laminin subunit gamma-2 | LAMC2 | Q13753 | 1 | 80 | [1] PMID: 24530674; [2] PMID: 19593942 |
| ECM remodelling and fibrosis | Periostin | POSTN | Q15063 | 1 | 80 | [1] PMID: 26498937 |
| ECM remodelling and fibrosis | Mothers against decapentaplegic homolog 2 | SMAD2 | Q15796 | 1 | 80 | [1] PMID: 22882904 |
| ECM remodelling and fibrosis | Laminin subunit alpha-4 | LAMA4 | Q16363 | 1 | 80 | [1] PMID: 24530674; [2] PMID: 19593942 |
| ECM remodelling and fibrosis | Laminin subunit alpha-3 | LAMA3 | Q16787 | 1 | 80 | [1] PMID: 24530674; [2] PMID: 19593942 |
| ECM remodelling and fibrosis | E3 ubiquitin-protein ligase pellino homolog 1 | PELI1 | Q96FA3 | 1 | 85 | [1] PMID: 24442869 |
| ECM remodelling and fibrosis | Matrix metalloproteinase-28 | MMP28 | Q9H239 | -1 | 80 | [1] PMID: 24519465 |
| ECM remodelling and fibrosis | Reticulon-4 | RTN4 | Q9NQC3 | 1 | 85 | [1] PMID: 29795235 |
| ECM remodelling and fibrosis | Laminin subunit gamma-3 | LAMC3 | Q9Y6N6 | 1 | 80 | [1] PMID: 24072174; [2] PMID: 19593942 |
| Hypertrophy | Nuclear factor of activated T-cells, cytoplasmic 1 | NFATC1 | O95644 | 1 | 85 | [1] PMID: 16847152; [2] PMID: 24161931 |
| Hypertrophy | Epidermal growth factor receptor | EGFR | P00533 | 1 | 75 | [1] PMID: 24126173; [2] PMID: 20564207; [3] PMID: 23909633 |
| Hypertrophy | Proto-oncogene c-Fos | FOS | P01100 | 1 | 75 | [1] PMID: 10869273 |
| Hypertrophy | Myc proto-oncogene protein | MYC | P01106 | 1 | 75 | [1] PMID: 10869273 |
| Hypertrophy | GTPase NRas | NRAS | P01111 | 1 | 75 | [1] PMID: 32007997 |
| Hypertrophy | GTPase HRas | HRAS | P01112 | 1 | 75 | [1] PMID: 32007997 |
| Hypertrophy | GTPase KRas | KRAS | P01116 | 1 | 75 | [1] PMID: 32007997 |
| Hypertrophy | Transforming growth factor beta-1 proprotein | TGFB1 | P01137 | 1 | 75 | [1] PMID: 10869273 |
| Hypertrophy | Natriuretic peptides A | NPPA | P01160 | 1 | 75 | [1] PMID: 10869273 |
| Hypertrophy | Somatotropin | GH1 | P01241 | 1 | 85 | [1] PMID: 3201924 |
| Hypertrophy | Interleukin-1 alpha | IL1A | P01583 | 1 | 85 | [1] PMID: 21148594 |
| Hypertrophy | Interstitial collagenase | MMP1 | P03956 | 1 | 75 | [1] PMID: 24126173; [2] PMID: 20564207 |
| Hypertrophy | Insulin-like growth factor I | IGF1 | P05019 | 1 | 85 | [1] PMID: 3201924 |
| Hypertrophy | Endothelin-1 | EDN1 | P05305 | 1 | 75 | [1] PMID: 10869273 |
| Hypertrophy | Transcription factor AP-1 | JUN | P05412 | 1 | 75 | [1] PMID: 10869273 |
| Hypertrophy | Gelsolin | GSN | P06396 | 1 | 75 | [1] PMID: 24505034; [2] PMID: 19246681; [3] PMID: 11120693 |
| Hypertrophy | Mineralocorticoid receptor | NR3C2 | P08235 | 1 | 75 | [1] PMID: 24126173; [2] PMID: 20564207 |
| Hypertrophy | Beta-1 adrenergic receptor | ADRB1 | P08588 | 1 | 75 | [1] PMID: 16750145 |
| Hypertrophy | Myosin light chain 3 | MYL3 | P08590 | 1 | 75 | [1] PMID: 10869273 |
| Hypertrophy | Fibroblast growth factor 2 | FGF2 | P09038 | 1 | 75 | [1] PMID: 24827991 |
| Hypertrophy | Myosin regulatory light chain 2, ventricular/cardiac muscle isoform | MYL2 | P10916 | 1 | 75 | [1] PMID: 10869273 |
| Hypertrophy | Bone morphogenetic protein 4 | BMP4 | P12644 | 1 | 80 | [1] PMID: 24736806 |
| Hypertrophy | Angiotensin-converting enzyme | ACE | P12821 | 1 | 75 | [1] PMID: 10869273 |
| Hypertrophy | Serine/threonine-protein phosphatase 2B catalytic subunit beta isoform | PPP3CB | P16298 | 1 | 85 | [1] Uniprot; [2] PMID: 16847152; [3] PMID: 24161931; [4] PMID: 28007541 |
| Hypertrophy | Natriuretic peptides B | NPPB | P16860 | 1 | 75 | [1] PMID: 10869273 |
| Hypertrophy | Early growth response protein 1 | EGR1 | P18146 | 1 | 75 | [1] PMID: 10869273 |
| Hypertrophy | Sodium/hydrogen exchanger 1 | SLC9A1 | P19634 | 1 | 85 | [1] PMID: 23909633; [2] PMID: 24080184; [3] PMID: 23429007 |
| Hypertrophy | Beta-adrenergic receptor kinase 1 | GRK2 | P25098 | 1 | 75 | [1] PMID: 10869273 |
| Hypertrophy | Endothelin-1 receptor | EDNRA | P25101 | 1 | 75 | [1] PMID: 16750145 |
| Hypertrophy | Mitogen-activated protein kinase 3 | MAPK3 | P27361 | 1 | 75 | [1] PMID: 32007997 |
| Hypertrophy | Mitogen-activated protein kinase 1 | MAPK1 | P28482 | 1 | 75 | [1] PMID: 32007997 |
| Hypertrophy | Nitric oxide synthase, endothelial | NOS3 | P29474 | -1 | 85 | [1] Uniprot; [2] PMID: 25977452 |
| Hypertrophy | Type-1 angiotensin II receptor | AGTR1 | P30556 | 1 | 80 | [1] PMID: 21784127; [2] PMID: 15526239; [3] PMID: 10869273 |
| Hypertrophy | Sodium/calcium exchanger 1 | SLC8A1 | P32418 | 1 | 80 | [1] PMID: 23909633; [2] PMID: 23506867; [3] PMID: 11922890 |
| Hypertrophy | Beta-adrenergic receptor kinase 2 | GRK3 | P35626 | 1 | 75 | [1] PMID: 10869273 |
| Hypertrophy | Nuclear receptor subfamily 2 group C member 2 | NR2C2 | P49116 | -1 | 75 | [1] PMID: 25752645; [2] PMID: 26564789 |
| Hypertrophy | Ribosomal protein S6 kinase alpha-3 | RPS6KA3 | P51812 | 1 | 80 | [1] Uniprot; [2] PMID: 23909633; [3] PMID: 30813401 |
| Hypertrophy | Caveolin-3 | CAV3 | P56539 | -1 | 75 | [1] PMID: 32007997 |
| Hypertrophy | Heart- and neural crest derivatives-expressed protein 2 | HAND2 | P61296 | 1 | 85 | [1] PMID: 16847152; [2] PMID: 24161931 |
| Hypertrophy | Calcineurin subunit B type 1 | PPP3R1 | P63098 | 1 | 75 | [1] Uniprot; [2] PMID: 16847152; [3] PMID: 24161931; [4] PMID: 18585734; [5] DOI: 10.1016/S1885-5857(06)60796-2 |
| Hypertrophy | Actin, alpha cardiac muscle 1 | ACTC1 | P68032 | 1 | 75 | [1] PMID: 10869273 |
| Hypertrophy | Myocyte-specific enhancer factor 2A | MEF2A | Q02078 | 1 | 85 | [1] PMID: 16847152; [2] PMID: 24161931 |
| Hypertrophy | Nuclear factor of activated T-cells, cytoplasmic 3 | NFATC3 | Q12968 | 1 | 85 | [1] PMID: 16847152; [2] PMID: 24161931 |
| Hypertrophy | Nuclear factor of activated T-cells, cytoplasmic 2 | NFATC2 | Q13469 | 1 | 85 | [1] PMID: 16847152; [2] PMID: 24161931 |
| Hypertrophy | Inositol 1,4,5-trisphosphate receptor type 2 | ITPR2 | Q14571 | 1 | 75 | [1] PMID: 21784127; [2] PMID: 24415751; [3] DOI: 10.1016/S1885-5857(06)60796-2 |
| Hypertrophy | Nuclear factor of activated T-cells, cytoplasmic 4 | NFATC4 | Q14934 | 1 | 85 | [1] PMID: 16847152; [2] PMID: 24161931 |
| Hypertrophy | Ribosomal protein S6 kinase alpha-2 | RPS6KA2 | Q15349 | 1 | 80 | [1] Uniprot; [2] PMID: 23909633; [3] PMID: 30813401 |
| Hypertrophy | Ribosomal protein S6 kinase alpha-1 | RPS6KA1 | Q15418 | 1 | 80 | [1] Uniprot; [2] PMID: 23909633; [3] PMID: 30813401 |
| Hypertrophy | Calcineurin subunit B type 2 | PPP3R2 | Q96LZ3 | 1 | 75 | [1] Uniprot; [2] PMID: 16847152; [3] PMID: 24161931; [4] PMID: 18585734; [5] DOI: 10.1016/S1885-5857(06)60796-2 |
| Hypertrophy | Peroxisome proliferator-activated receptor gamma coactivator 1-alpha | PPARGC1A | Q9UBK2 | -1 | 75 | [1] PMID: 21799152; [2] PMID: 26356605; [3] PMID: 20888832 |
| Hypertrophy | Short transient receptor potential channel 4 | TRPC4 | Q9UBN4 | -1 | 80 | [1] PMID: 25047165 |
| Cardiomyocyte cell death | HCLS1-associated protein X-1 | HAX1 | O00165 | -1 | 75 | [1] PMID: 26553996 |
| Cardiomyocyte cell death | Dynamin-1-like protein | DNM1L | O00429 | 1 | 80 | [1] PMID: 24905188 |
| Cardiomyocyte cell death | Mitogen-activated protein kinase 13 | MAPK13 | O15264 | 1 | 75 | [1] PMID: 23316291 |
| Cardiomyocyte cell death | Baculoviral IAP repeat-containing protein 5 | BIRC5 | O15392 | -1 | 75 | [1] PMID: 19919994 |
| Cardiomyocyte cell death | Serine protease HTRA2, mitochondrial | HTRA2 | O43464 | 1 | 75 | [1] PMID: 22535253 |
| Cardiomyocyte cell death | Serine/threonine-protein kinase ULK1 | ULK1 | O75385 | 1 | 80 | [1] PMID: 24859226 |
| Cardiomyocyte cell death | Mitofusin-2 | MFN2 | O95140 | -1 | 80 | [1] PMID: 24905188 |
| Cardiomyocyte cell death | Cellular tumor antigen p53 | TP53 | P04637 | 1 | 80 | [1] PMID: 14962485 |
| Cardiomyocyte cell death | ADP/ATP translocase 2 | SLC25A5 | P05141 | 1 | 75 | [1] PMID: 19179805; [2] PMID: 26553996 |
| Cardiomyocyte cell death | Apoptosis regulator Bcl-2 | BCL2 | P10415 | -1 | 80 | [1] PMID: 25663976 |
| Cardiomyocyte cell death | ADP/ATP translocase 1 | SLC25A4 | P12235 | 1 | 75 | [1] PMID: 19179805; [2] PMID: 26553996 |
| Cardiomyocyte cell death | ADP/ATP translocase 3 | SLC25A6 | P12236 | 1 | 75 | [1] PMID: 19179805; [2] PMID: 26553996 |
| Cardiomyocyte cell death | Bone morphogenetic protein 4 | BMP4 | P12644 | 1 | 80 | [1] PMID: 24736806 |
| Cardiomyocyte cell death | Lysosome-associated membrane glycoprotein 2 | LAMP2 | P13473 | -1 | 80 | [1] PMID: 24859226 |
| Cardiomyocyte cell death | Voltage-dependent anion-selective channel protein 1 | VDAC1 | P21796 | 1 | 75 | [1] PMID: 19179805; [2] PMID: 26553996 |
| Cardiomyocyte cell death | Tumor necrosis factor receptor superfamily member 6 | FAS | P25445 | 1 | 80 | [1] PMID: 25663976 |
| Cardiomyocyte cell death | Peptidyl-prolyl cis-trans isomerase F, mitochondrial | PPIF | P30405 | 1 | 80 | [1] PMID: 26553996 |
| Cardiomyocyte cell death | Mitogen-activated protein kinase 4 | MAPK4 | P31152 | 1 | 75 | [1] PMID: 23316291 |
| Cardiomyocyte cell death | Metalloproteinase inhibitor 3 | TIMP3 | P35625 | -1 | 80 | [1] PMID: 23316962 |
| Cardiomyocyte cell death | Serine/threonine-protein kinase mTOR | MTOR | P42345 | -1 | 80 | [1] PMID: 24859226 |
| Cardiomyocyte cell death | Caspase-3 | CASP3 | P42574 | 1 | 85 | [1] PMID: 25663976 |
| Cardiomyocyte cell death | Voltage-dependent anion-selective channel protein 2 | VDAC2 | P45880 | 1 | 75 | [1] PMID: 19179805; [2] PMID: 26553996 |
| Cardiomyocyte cell death | Tumor necrosis factor ligand superfamily member 6 | FASLG | P48023 | 1 | 80 | [1] PMID: 25663976 |
| Cardiomyocyte cell death | Tuberin | TSC2 | P49815 | 1 | 75 | [1] PMID: 24859226 |
| Cardiomyocyte cell death | Glycogen synthase kinase-3 alpha | GSK3A | P49840 | -1 | 75 | [1] PMID: 22086876 |
| Cardiomyocyte cell death | Glycogen synthase kinase-3 beta | GSK3B | P49841 | 1 | 75 | [1] PMID: 19179805; [2] PMID: 26553996 |
| Cardiomyocyte cell death | Mitogen-activated protein kinase 12 | MAPK12 | P53778 | 1 | 75 | [1] PMID: 23316291 |
| Cardiomyocyte cell death | Caspase-9 | CASP9 | P55211 | 1 | 85 | [1] PMID: 25717353 |
| Cardiomyocyte cell death | BH3-interacting domain death agonist | BID | P55957 | 1 | 80 | [1] PMID: 17657164 |
| Cardiomyocyte cell death | E3 ubiquitin-protein ligase XIAP | XIAP | P98170 | -1 | 75 | [1] PMID: 19919994 |
| Cardiomyocyte cell death | Apoptosis regulator BAX | BAX | Q07812 | 1 | 80 | [1] PMID: 25520329 |
| Cardiomyocyte cell death | Induced myeloid leukemia cell differentiation protein Mcl-1 | MCL1 | Q07820 | -1 | 75 | [1] PMID: 17490677 |
| Cardiomyocyte cell death | BCL2/adenovirus E1B 19 kDa protein-interacting protein 3 | BNIP3 | Q12983 | 1 | 75 | [1] PMID: 24859226 |
| Cardiomyocyte cell death | Baculoviral IAP repeat-containing protein 2 | BIRC2 | Q13490 | -1 | 75 | [1] PMID: 19919994 |
| Cardiomyocyte cell death | Beclin-1 | BECN1 | Q14457 | 1 | 80 | [1] PMID: 24859226 |
| Cardiomyocyte cell death | Caspase-8 | CASP8 | Q14790 | 1 | 80 | [1] PMID: 25663976 |
| Cardiomyocyte cell death | Mitogen-activated protein kinase 11 | MAPK11 | Q15759 | 1 | 75 | [1] PMID: 23316291 |
| Cardiomyocyte cell death | Mitogen-activated protein kinase 14 | MAPK14 | Q16539 | 1 | 75 | [1] PMID: 23316291 |
| Cardiomyocyte cell death | Mitogen-activated protein kinase 6 | MAPK6 | Q16659 | 1 | 75 | [1] PMID: 23316291 |
| Cardiomyocyte cell death | Mitofusin-1 | MFN1 | Q8IWA4 | -1 | 85 | [1] PMID: 24615014 |
| Cardiomyocyte cell death | Regulatory-associated protein of mTOR | RPTOR | Q8N122 | 1 | 75 | [1] PMID: 24859226 |
| Cardiomyocyte cell death | Bcl2-associated agonist of cell death | BAD | Q92934 | 1 | 75 | [1] PMID: 17490677 |
| Cardiomyocyte cell death | Mitogen-activated protein kinase kinase kinase 5 | MAP3K5 / ASK1 | Q99683 | 1 | 80 | [1] PMID: 23316291 |
| Cardiomyocyte cell death | ADP/ATP translocase 4 | SLC25A31 | Q9H0C2 | 1 | 75 | [1] PMID: 19179805; [2] PMID: 26553996 |
| Cardiomyocyte cell death | Autophagy protein 5 | ATG5 | Q9H1Y0 | -1 | 80 | [1] PMID: 24859226 |
| Cardiomyocyte cell death | Reticulon-4 | RTN4 | Q9NQC3 | 1 | 85 | [1] PMID: 29795235 |
| Cardiomyocyte cell death | Diablo homolog, mitochondrial | DIABLO | Q9NR28 | 1 | 75 | [1] PMID: 14998631 |
| Cardiomyocyte cell death | NAD-dependent protein deacetylase sirtuin-3, mitochondrial | SIRT3 | Q9NTG7 | -1 | 85 | [1] PMID: 30219671 |
| Cardiomyocyte cell death | Voltage-dependent anion-selective channel protein 3 | VDAC3 | Q9Y277 | 1 | 75 | [1] PMID: 19179805; [2] PMID: 26553996 |
| Impaired myocyte contractility | Voltage-dependent L-type calcium channel subunit beta-4 | CACNB4 | O00305 | -1 | 75 | [1] PMID: 18981739 |
| Impaired myocyte contractility | cGMP-dependent 3',5'-cyclic phosphodiesterase | PDE2A | O00408 | -1 | 80 | [1] PMID: 27799254 |
| Impaired myocyte contractility | Sarcoplasmic/endoplasmic reticulum calcium ATPase 1 | ATP2A1 | O14983 | 1 | 80 | [1] PMID: 18981739 |
| Impaired myocyte contractility | Voltage-dependent L-type calcium channel subunit alpha-1F | CACNA1F | O60840 | -1 | 75 | [1] PMID: 18981739 |
| Impaired myocyte contractility | Guanylate cyclase soluble subunit beta-2 | GUCY1B2 | O75343 | -1 | 80 | [1] PMID: 25977452 |
| Impaired myocyte contractility | Transforming growth factor beta-1 proprotein | TGFB1 | P01137 | 1 | 80 | [1] PMID: 25788886 |
| Impaired myocyte contractility | Collagen alpha-1(I) chain | COL1A1 | P02452 | 1 | 80 | [1] PMID: 12031701 |
| Impaired myocyte contractility | Estrogen receptor | ESR1 | P03372 | -1 | 80 | [1] PMID: 19893013 |
| Impaired myocyte contractility | Insulin-like growth factor I | IGF1 | P05019 | -1 | 75 | [1] PMID: 10591031 |
| Impaired myocyte contractility | Calpain-1 catalytic subunit | CAPN1 | P07384 | 1 | 85 | [1] PMID: 24891510 |
| Impaired myocyte contractility | Beta-1 adrenergic receptor | ADRB1 | P08588 | 1 | 80 | [1] PMID: 19633206 |
| Impaired myocyte contractility | Thyroid hormone receptor alpha | THRA | P10827 | -1 | 90 | [1] PMID: 17379774 |
| Impaired myocyte contractility | Thyroid hormone receptor beta | THRB | P10828 | -1 | 90 | [1] PMID: 17379774 |
| Impaired myocyte contractility | Myosin-7 | MYH7 | P12883 | 1 | 90 | [1] PMID: 17379774 |
| Impaired myocyte contractility | Myosin-6 | MYH6 | P13533 | -1 | 90 | [1] PMID: 19679836; [2] PMID: 17379774 |
| Impaired myocyte contractility | Serine/threonine-protein phosphatase 2B catalytic subunit beta isoform | PPP3CB | P16298 | 1 | 75 | [1] Uniprot; [2] PMID: 16847152 |
| Impaired myocyte contractility | Sarcoplasmic/endoplasmic reticulum calcium ATPase 2 | ATP2A2 | P16615 | 1 | 80 | [1] PMID: 18981739 |
| Impaired myocyte contractility | cAMP-dependent protein kinase catalytic subunit alpha | PRKACA | P17612 | 1 | 80 | [1] PMID: 18981739 |
| Impaired myocyte contractility | cAMP-dependent protein kinase catalytic subunit gamma | PRKACG | P22612 | 1 | 80 | [1] PMID: 18981739 |
| Impaired myocyte contractility | cAMP-dependent protein kinase catalytic subunit beta | PRKACB | P22694 | 1 | 80 | [1] PMID: 18981739 |
| Impaired myocyte contractility | Cardiac phospholamban | PLN | P26678 | -1 | 80 | [1] PMID: 18981739 |
| Impaired myocyte contractility | Nitric oxide synthase, endothelial | NOS3 | P29474 | -1 | 85 | [1] Uniprot; [2] PMID: 25977452 |
| Impaired myocyte contractility | Guanylate cyclase soluble subunit alpha-2 | GUCY1A2 | P33402 | -1 | 80 | [1] PMID: 25977452 |
| Impaired myocyte contractility | Alpha-1A adrenergic receptor | ADRA1A | P35348 | -1 | 75 | [1] PMID: 16859660 |
| Impaired myocyte contractility | Serine/threonine-protein phosphatase 2B catalytic subunit gamma isoform | PPP3CC | P48454 | 1 | 75 | [1] Uniprot; [2] PMID: 16847152 |
| Impaired myocyte contractility | CX3C chemokine receptor 1 | CX3CR1 | P49238 | 1 | 80 | [1] PMID: 18585734 |
| Impaired myocyte contractility | Beta-arrestin-1 | ARRB1 | P49407 | 1 | 80 | [1] PMID: 24218435 |
| Impaired myocyte contractility | Calcineurin subunit B type 1 | PPP3R1 | P63098 | 1 | 85 | [1] Uniprot; [2] PMID: 16847152 |
| Impaired myocyte contractility | Fractalkine | CX3CL1 | P78423 | 1 | 80 | [1] PMID: 18585734 |
| Impaired myocyte contractility | Voltage-dependent L-type calcium channel subunit alpha-1D | CACNA1D | Q01668 | -1 | 75 | [1] PMID: 18981739 |
| Impaired myocyte contractility | Myocyte-specific enhancer factor 2A | MEF2A | Q02078 | 1 | 85 | [1] PMID: 16847152 |
| Impaired myocyte contractility | Myocyte-specific enhancer factor 2B | MEF2B | Q02080 | 1 | 75 | [1] PMID: 16847152 |
| Impaired myocyte contractility | Guanylate cyclase soluble subunit alpha-1 | GUCY1A1 | Q02108 | -1 | 80 | [1] PMID: 25977452 |
| Impaired myocyte contractility | Guanylate cyclase soluble subunit beta-1 | GUCY1B1 | Q02153 | -1 | 80 | [1] PMID: 25977452 |
| Impaired myocyte contractility | Voltage-dependent L-type calcium channel subunit beta-1 | CACNB1 | Q02641 | -1 | 75 | [1] PMID: 18981739 |
| Impaired myocyte contractility | Myocyte-specific enhancer factor 2C | MEF2C | Q06413 | 1 | 75 | [1] PMID: 16847152 |
| Impaired myocyte contractility | Serine/threonine-protein phosphatase 2B catalytic subunit alpha isoform | PPP3CA | Q08209 | 1 | 75 | [1] Uniprot; [2] PMID: 16847152 |
| Impaired myocyte contractility | Voltage-dependent L-type calcium channel subunit beta-2 | CACNB2 | Q08289 | -1 | 75 | [1] PMID: 18981739 |
| Impaired myocyte contractility | cGMP-dependent protein kinase 2 | PRKG2 | Q13237 | -1 | 85 | [1] Uniprot; [2] PMID: 25977452 |
| Impaired myocyte contractility | Calcium/calmodulin-dependent protein kinase type II subunit beta | CAMK2B | Q13554 | 1 | 75 | [1] PMID: 15793582 |
| Impaired myocyte contractility | Calcium/calmodulin-dependent protein kinase type II subunit gamma | CAMK2G | Q13555 | 1 | 75 | [1] PMID: 15793582 |
| Impaired myocyte contractility | Calcium/calmodulin-dependent protein kinase type II subunit delta | CAMK2D | Q13557 | 1 | 75 | [1] PMID: 15793582 |
| Impaired myocyte contractility | Voltage-dependent L-type calcium channel subunit alpha-1S | CACNA1S | Q13698 | -1 | 75 | [1] PMID: 18981739 |
| Impaired myocyte contractility | Voltage-dependent L-type calcium channel subunit alpha-1C | CACNA1C | Q13936 | -1 | 75 | [1] PMID: 18981739 |
| Impaired myocyte contractility | cGMP-dependent protein kinase 1 | PRKG1 | Q13976 | -1 | 85 | [1] Uniprot; [2] PMID: 25977452 |
| Impaired myocyte contractility | Myocyte-specific enhancer factor 2D | MEF2D | Q14814 | 1 | 85 | [1] PMID: 16847152 |
| Impaired myocyte contractility | Titin | TTN | Q8WZ42 | -1 | 85 | [1] PMID: 25977452 |
| Impaired myocyte contractility | Sarcoplasmic/endoplasmic reticulum calcium ATPase 3 | ATP2A3 | Q93084 | 1 | 80 | [1] PMID: 18981739 |
| Impaired myocyte contractility | Calcineurin subunit B type 2 | PPP3R2 | Q96LZ3 | 1 | 85 | [1] Uniprot; [2] PMID: 16847152 |
| Impaired myocyte contractility | Histone deacetylase 9 | HDAC9 | Q9UKV0 | 1 | 75 | [1] PMID: 19893013 |
| Impaired myocyte contractility | Histone deacetylase 5 | HDAC5 | Q9UQL6 | 1 | 75 | [1] PMID: 19893013 |
| Impaired myocyte contractility | Calcium/calmodulin-dependent protein kinase type II subunit alpha | CAMK2A | Q9UQM7 | 1 | 75 | [1] PMID: 15793582 |
| Inflammation | Complement C1q and tumor necrosis factor-related protein 9B | C1QTNF9B | B2RNN3 | -1 | 85 | [1] PMID: 31623508 |
| Inflammation | Toll-like receptor 4 | TLR4 | O00206 | 1 | 90 | [1] PMID: 31623508 |
| Inflammation | Interleukin-33 | IL33 | O95760 | -1 | 80 | [1] PMID: 32432676 |
| Inflammation | Transforming growth factor beta-1 proprotein | TGFB1 | P01137 | -1 | 85 | [1] PMID: 26498937; [2] PMID: 17109837 |
| Inflammation | Tumor necrosis factor | TNF | P01375 | 1 | 90 | [1] PMID: 28173864 |
| Inflammation | Interferon gamma | IFNG | P01579 | 1 | 85 | [1] PMID: 25583678 |
| Inflammation | Interleukin-1 alpha | IL1A | P01583 | 1 | 90 | [1] PMID: 25583678; [2] PMID: 23806284 |
| Inflammation | Interleukin-1 beta | IL1B | P01584 | 1 | 90 | [1] PMID: 31623508; [2] PMID: 23806284 |
| Inflammation | C-reactive protein | CRP | P02741 | 1 | 85 | [1] PMID: 25583678 |
| Inflammation | Myeloperoxidase | MPO | P05164 | 1 | 80 | [1] PMID: 22260952 |
| Inflammation | Interleukin-6 | IL6 | P05231 | 1 | 90 | [1] PMID: 31623508 |
| Inflammation | Intercellular adhesion molecule 1 | ICAM1 | P05362 | 1 | 85 | [1] PMID: 17346172 |
| Inflammation | Growth-regulated alpha protein | CXCL1 | P09341 | 1 | 85 | [1] PMID: 23925450 |
| Inflammation | Complement C1q and tumor necrosis factor-related protein 9A | C1QTNF9 | P0C862 | -1 | 85 | [1] PMID: 31623508 |
| Inflammation | Interleukin-8 | CXCL8 | P10145 | 1 | 90 | [1] PMID: 26498937 |
| Inflammation | C-C motif chemokine 2 | CCL2 | P13500 | 1 | 90 | [1] PMID: 26498937 |
| Inflammation | C-C motif chemokine 5 | CCL5 | P13501 | 1 | 85 | [1] PMID: 23925450 |
| Inflammation | P-selectin | SELP | P16109 | 1 | 85 | [1] PMID: 30873166 |
| Inflammation | E-selectin | SELE | P16581 | 1 | 85 | [1] PMID: 30873166 |
| Inflammation | C-X-C motif chemokine 2 | CXCL2 | P19875 | 1 | 90 | [1] PMID: 26498937 |
| Inflammation | Interleukin-10 | IL10 | P22301 | -1 | 85 | [1] PMID: 26498937 |
| Inflammation | Atypical chemokine receptor 3 | ACKR3 | P25106 | 1 | 85 | [1] PMID: 32170348 |
| Inflammation | Tumor necrosis factor receptor superfamily member 6 | FAS | P25445 | 1 | 85 | [1] PMID: 23176689 |
| Inflammation | Mitogen-activated protein kinase 3 | MAPK3 | P27361 | 1 | 85 | [1] PMID: 32170348 |
| Inflammation | Mitogen-activated protein kinase 1 | MAPK1 | P28482 | 1 | 85 | [1] PMID: 32170348 |
| Inflammation | Caspase-1 | CASP1 | P29466 | 1 | 80 | [1] PMID: 24530674 |
| Inflammation | Tumor necrosis factor ligand superfamily member 10 | TNFSF10 | P50591 | -1 | 80 | [1] PMID: 23176689 |
| Inflammation | Eotaxin | CCL11 | P51671 | 1 | 85 | [1] PMID: 23925450 |
| Inflammation | Transcription factor p65 | RELA | Q04206 | 1 | 90 | [1] PMID: 31623508 |
| Inflammation | FAS-associated death domain protein | FADD | Q13158 | 1 | 80 | [1] PMID: 23176689 |
| Inflammation | Tumor necrosis factor receptor type 1-associated DEATH domain protein | TRADD | Q15628 | 1 | 80 | [1] PMID: 23176689 |
| Inflammation | Myeloid differentiation primary response protein MyD88 | MYD88 | Q99836 | 1 | 80 | [1] PMID: 23806284; [2] PMID: 20125030 |
| Inflammation | Growth/differentiation factor 15 | GDF15 | Q99988 | -1 | 80 | [1] PMID: 22260952 |
| RAS activation | Vascular endothelial growth factor D | VEGFD | O43915 | 1 | 80 | [1] PMID: 28656296 |
| RAS activation | Renin | REN | P00797 | 1 | 85 | [1] PMID: 22260952 |
| RAS activation | Angiotensinogen | AGT | P01019 | 1 | 95 | [1] PMID: 28656296 |
| RAS activation | Platelet-derived growth factor subunit B | PDGFB | P01127 | 1 | 80 | [1] doi: 10.1007/978-3-642-18497-0_12 |
| RAS activation | Transforming growth factor beta-1 proprotein | TGFB1 | P01137 | 1 | 85 | [1] PMID: 25717353 |
| RAS activation | Natriuretic peptides A | NPPA | P01160 | 1 | 80 | [1] PMID: 10506489 |
| RAS activation | Platelet-derived growth factor subunit A | PDGFA | P04085 | 1 | 80 | [1] doi: 10.1007/978-3-642-18497-0_12 |
| RAS activation | Cellular tumor antigen p53 | TP53 | P04637 | 1 | 75 | [1] PMID: 10506489 |
| RAS activation | Cytochrome b-245 heavy chain | CYBB | P04839 | 1 | 85 | [1] doi:10.1038/labinvest.2012.136 |
| RAS activation | Insulin-like growth factor I | IGF1 | P05019 | 1 | 80 | [1] doi: 10.1007/978-3-642-18497-0_12 |
| RAS activation | Cathepsin D | CTSD | P07339 | 1 | 80 | [1] PMID: 10506489 |
| RAS activation | Beta-2 adrenergic receptor | ADRB2 | P07550 | 1 | 80 | [1] PMID: 10506489 |
| RAS activation | Beta-1 adrenergic receptor | ADRB1 | P08588 | 1 | 80 | [1] PMID: 10506489 |
| RAS activation | Angiotensin-converting enzyme | ACE | P12821 | 1 | 85 | [1] PMID: 10869273 |
| RAS activation | C-C motif chemokine 2 | CCL2 | P13500 | 1 | 85 | [1] doi:10.1038/labinvest.2012.136 |
| RAS activation | Matrix metalloproteinase-9 | MMP9 | P14780 | 1 | 80 | [1] PMID: 28656296 |
| RAS activation | Type-1 angiotensin II receptor | AGTR1 | P30556 | 1 | 85 | [1] PMID: 22260952 |
| RAS activation | Mas-related G-protein coupled receptor MRG | MAS1L | P35410 | 1 | 95 | [1] PMID: 28656296 |
| RAS activation | Signal transducer and activator of transcription 3 | STAT3 | P40763 | 1 | 75 | [1] PMID: 10506489 |
| RAS activation | Signal transducer and activator of transcription 6 | STAT6 | P42226 | 1 | 75 | [1] PMID: 10506489 |
| RAS activation | Type-2 angiotensin II receptor | AGTR2 | P50052 | 1 | 85 | [1] PMID: 22260952 |
| RAS activation | Angiotensin-converting enzyme 2 | ACE2 | Q9BYF1 | 1 | 95 | [1] PMID: 28656296 |
| RAS activation | NADPH oxidase 4 | NOX4 | Q9NPH5 | 1 | 85 | [1] doi:10.1038/labinvest.2012.136 |

ECM, extracellular matrix; RAS, renin-angiotensin system.

**Supplemental Table 2. Pirfenidone targets and bioflags.**

| **Targets** |  |  |  |  |
| --- | --- | --- | --- | --- |
| **UniProt ID** | **Protein Name** | **Gene name** | **Effect** | **References** |
| P09958 | Furin | FURIN (Furin) | Inhibition | [1] PMID: 17234158 |
| P05121 | Plasminogen activator inhibitor 1 | SERPINE (PAI-1) | Activation | [1] PMID: **31693876**; [2] PMID: 23002925 |
| P53778 | Mitogen-activated protein kinase 12 | MAPK12 (p38𝛾) | Inhibition | [1] PMID: **30971822** |
| P10070 | Zinc finger protein GLI2 | GLI2 (GLI2) | Inhibition | [1] PMID: **28148565**; [2] PMID: **31036756** |
|  | | | | |
| **Bioflags** | | | | |
| P09237 | Matrix metalloproteinase-7 (Matrilysin) | MMP7 | Inhibition | [1] PMID: **30072107**; [2] PMID: **32448163** |
| P49841 | Glycogen synthase kinase-3 beta | GSK3B | Inhibition | [1] PMID: **28537766** |
| P05412 | Transcription factor AP-1 | JUN | Inhibition | [1] PMID: **27615411** |
| P01579 | Interferon gamma | IFNG | Inhibition | [1] PMID: **31967851** |
| P01375 | Tumor necrosis factor | TNF | Inhibition | [1] PMID: **31777533**; [2] PMID: **32381456** |
| P12821 | Angiotensin-converting enzyme | ACE | Inhibition | [1] PMID: **32575019** |
| P30556 | Type-1 angiotensin II receptor | AGTR1 | Inhibition | [1] PMID: **32575019** |
| P02751 | Fibronectin | FN1 | Inhibition | [1] PMID: **31967851**; [2] PMID: **27245114** |
| P42226 | Signal transducer and activator of transcription 6 | STAT6 | Inhibition | [1] PMID: **27788604** |
| P08254 | Stromelysin-1 | MMP3 | Inhibition | [1] PMID: **27181436** |
| Q9NPH5 | NADPH oxidase 4 | NOX4 | Inhibition | [1] PMID: **32587469** |
| P09038 | Fibroblast growth factor 2 | FGF2 | Inhibition |  |
| P04085 | Platelet-derived growth factor subunit A | PDGFA | Inhibition | [1] PMID: **31967851** |
| P01127 | Platelet-derived growth factor subunit B | PDGFB | Inhibition | [1] PMID: **31967851** |
| Q9H1Y0 | Autophagy protein 5 | ATG5 | Activation | [1] PMID: **28577568** |
| O95352 | Ubiquitin-like modifier-activating enzyme ATG7 | ATG7 | Activation | [1] PMID: **28577568** |
| P40763 | Signal transducer and activator of transcription 3 | STAT3 | Inhibition | [1] PMID: **30481203** |
| P01137 | Transforming growth factor beta-1 proprotein | TGFB1 | Inhibition | [1] PMID: **22222821**; [2] PMID: **29374548** |
| P32418 | Sodium/calcium exchanger 1 | SLC8A1 | Inhibition | [1] PMID: **30618813** |
| Q9UPR5 | Sodium/calcium exchanger 2 | SLC8A2 | Inhibition | [1] PMID: **30618813** |
| P57103 | Sodium/calcium exchanger 3 | SLC8A3 | Inhibition | [1] PMID: **30618813** |

**Supplemental Table 3. List of proteins expected to mediate the effects of pirfenidone on post-myocardial infarction remodeling.**

| **Protein name** | **Gene name** | **Uniprot code** |
| --- | --- | --- |
| Disintegrin and metalloproteinase domain-containing protein 17 | TACE | P78536 |
| Androgen receptor | AR | P10275 |
| Apoptosis regulator BAX | BAX | Q07812 |
| Apoptosis regulator Bcl-2 | BCL2 | P10415 |
| C-X-C chemokine receptor type 4 | CXCR4 | P61073 |
| Endothelin-1 | EDN1 | P05305 |
| Early growth response protein 1 | EGR1 | P18146 |
| Proto-oncogene c-Fos | FOS | P01100 |
| Forkhead box protein O1 | FOXO1 | Q12778 |
| Forkhead box protein O3 | FOXO3 | O43524 |
| Furin | FURIN | P09958 |
| Interferon gamma | IFNG | P01579 |
| Insulin-like growth factor I | IGF1 | P05019 |
| Transcription factor AP-1 | JUN | P05412 |
| Mitogen-activated protein kinase 12 | MAPK12 | P53778 |
| Matrix metalloproteinase-14 | MMP14 | P50281 |
| 72 kDa type IV collagenase | MMP2 | P08253 |
| Sodium/calcium exchanger 1 | SLC8A1 | P32418 |
| Nuclear factor of activated T-cells, cytoplasmic 1 | NFATC1 | O95644 |
| Nuclear factor of activated T-cells, cytoplasmic 2 | NFATC2 | Q13469 |
| Nuclear factor of activated T-cells, cytoplasmic 3 | NFATC3 | Q12968 |
| Nuclear factor NF-kappa-B p105 subunit | NFKB1 | P19838 |
| Platelet-derived growth factor subunit A | PDGFA | P04085 |
| Platelet-derived growth factor subunit B | PDGFB | P01127 |
| Mothers against decapentaplegic homolog 2 | SMAD2 | Q15796 |
| Mothers against decapentaplegic homolog 3 | SMAD3 | P84022 |
| Transforming growth factor beta-1 proprotein | TGFB1 | P01137 |
| Tumor necrosis factor | TNFA | P01375 |
| Vitamin D3 receptor | VDR | P11473 |
| Disintegrin and metalloproteinase domain-containing protein 17 | TACE | P78536 |
